# Impact of sedation with propofol and/or sufentanil on short-term outcomes in patients admitted to the intensive care unit: A retrospective study

**DOI:** 10.1177/03000605261461651

**Published:** 2026-08-10

**Authors:** Luca Scarsella, Contrino Rita, El-Masri Dschamil, Thal Serge C

**Affiliations:** Helios University Hospital Wuppertal, Department of Anesthesiology, Germany

**Keywords:** Sedation, propofol, sufentanil, intensive care unit, Sequential Organ Failure Assessment score, reintubation

## Abstract

**Background:**

In the intensive care unit, sedation plays a crucial role in managing critically ill patients, particularly those undergoing invasive procedures. Common sedatives such as propofol and sufentanil are administered to ensure comfort and alleviate pain among intensive care unit patients. However, prolonged sedation is associated with risks, including delayed recovery, increased susceptibility to infections, and challenges in monitoring critical conditions. This study investigated the effect of sedation using only propofol, only sufentanil, and a combination of both on short-term outcomes in intensive care unit patients.

**Methods:**

In total, 300 adult intensive care unit patients, both postoperative and nonpostoperative, were included in this retrospective study. The observation period was 10 days from intensive care unit admission. In addition to baseline clinical data, intensive care unit length of stay, delta Sequential Organ Failure Assessment score, occurrence of pulmonary infiltrates on chest X-ray, onset of acute kidney injury, and need for reintubation were assessed as study outcomes.

**Results:**

Results showed that patients sedated using sufentanil alone or in combination with propofol had a longer length of intensive care unit stay and exhibited higher delta Sequential Organ Failure Assessment scores, which worsened over a 10-day period compared with those who were not sedated or administered propofol alone. Additionally, new pulmonary infiltrates occurred more frequently in patients sedated with a combination of propofol and sufentanil than in those who were administered propofol alone or no sedation. Patients who were administered sufentanil alone were reintubated more often than those who were not sedated or were administered propofol alone.

**Conclusions:**

The use of sufentanil, alone or combined with propofol, is associated with poorer short-term outcomes in ICU patients. These findings highlight the need for careful evaluation of sedation strategies in critically ill patients.

## Introduction

In the intensive care unit (ICU), sedation is a critical aspect of patient management, especially for those undergoing invasive procedures, experiencing significant pain, or requiring mechanical ventilation.^1^ The primary goals of sedation are to ensure patient comfort, reduce anxiety, alleviate pain, prevent agitation, facilitate medical interventions, and ensure patient safety.^2^ Sedation practices in the ICU are highly individualized, based on the patient's condition, level of consciousness, and type of medical interventions required.^1,3–5^ Sedation in the ICU involves the use of several classes of drugs, each serving different purposes and offering distinct advantages. Sufentanil and propofol are two commonly used sedative agents in the ICU setting.^6^ Sufentanil, an analogue of fentanyl, is a potent synthetic opioid analgesic, with high selectivity for the µ-opioid receptor. It is 625–4000 times more potent than morphine and 5–15 times more potent than fentanyl.^7–9^ Sufentanil is highly liposoluble, resulting in a faster passage through the blood–brain barrier, with remarkable sedative properties.^7–10^ Sufentanil can be an effective sedative and analgesic for ICU patients, especially those requiring mechanical ventilation or undergoing invasive procedures.^9^ However, it must be used cautiously owing to the associated risk of respiratory depression, hypotension, and potential for opioid-related adverse effects.^8^ Propofol is also a widely used sedative in the ICU setting for patients who require mechanical ventilation or are undergoing invasive procedures.^11^ It is a short-acting intravenous anesthetic known for its rapid onset, ease of titration, and relatively quick recovery time. Propofol acts by enhancing the activity of gamma-aminobutyric acid (GABA), an inhibitory neurotransmitter, leading to central nervous system depression, resulting in sedation and anterograde amnesia.^11,12^ Propofol is an effective and versatile sedative in the ICU; however, it requires careful monitoring because its use is associated with the risks of hypotension, respiratory depression, and propofol infusion syndrome.^13^

Prolonged sedation involves significant risks, including delayed recovery, increased risk of infections (such as ventilator-associated pneumonia), and organ dysfunction, potentially leading to failure.^14^ It often arises in ICU settings due to increased patient vulnerability. Recent scientific evidence has shown that light sedation is associated with a shorter ICU stay than deeper sedation.^15^ Although the use of propofol as a sedative in ICU patients has been shown to be safe and not associated with adverse outcomes in patients with sepsis, opioids are generally observed to exert an immunosuppressive effect.^16,17^ In the present study, we analyzed the effect of sedation with propofol alone, sufentanil alone, and a combination of the two on short-term outcomes in adult ICU patients by examining ICU stay duration, progression of the delta Sequential Organ Failure Assessment (SOFA) score, reintubation rate, incidence of new pulmonary infiltrates, and onset of acute kidney injury (AKI) (in accordance to the Kidney Disease: Improving Global Outcomes (KDIGO) guideline^18^). Based on the described immunosuppressive effects of opioids, we hypothesize that the presence of opioids within a sedation regimen, even when used in combination with other sedatives, is associated with worse clinical outcomes compared with the use of propofol for sedation or no sedation.

## Patients and methods

### Design and setting

We conducted a retrospective analysis of the medical records of 300 patients admitted to the ICU of Helios University Hospital Wuppertal, Germany. Data were retrieved for patients admitted between 1 January 2021 and 31 December 2023. This study is a hypothesis-generating, monocentric and exploratory study.

### Patients

Patients were selected using the following inclusion criteria: (a) age ≥18 years and (b) ICU stay ≥1 day. The medical records of the included patients were subsequently screened for the following exclusion criteria: (a) age <18 years; (b) ICU stay <1 day; (c) pre-existing infections at admission; and (d) immunosuppression (pregnancy, chemotherapy, radiation therapy, HIV, and/or immunosuppressive medications). Both surgical patients (whose admission to the ICU was determined either by the invasiveness of the surgical procedure, preoperative health condition, or intraoperative course) and nonsurgical patients were included in the study. The study population was divided into 4 groups: (a) no sedation (patients admitted to the ICU who were not intubated or sedated); (b) propofol only (patients admitted to the ICU who may or may not be intubated and sedated using propofol alone for at least 24 h; sufentanil was never administered to this group); (c) sufentanil only (patients admitted to the ICU who may or may not be intubated and sedated using only sufentanil for at least 24 h; propofol was never administered to this group); (d) propofol plus sufentanil (patients admitted to the ICU who may or may not be intubated and sedated using propofol and sufentanil for at least 24 h; in some cases, this was preceded or followed by the administration of sufentanil alone or propofol alone.

### Data collection

We collected the following routine registration data from the ICU electronic medical records digitally using a pseudonymized digital data collection form: (a) core patient data (sex); (b) medical history data (presence of chronic kidney disease (CKD), chronic obstructive pulmonary disease (COPD), diabetes mellitus (DM)); (c) American Society of Anesthesiologists ASA) classification; (d) date of ICU admission and length of ICU stay; (e) ΔSOFA score; (f) time of reintubation; (g) and time of a new diagnosis of AKI during the 10-day stay in the ICU.

### Definitions of endpoints and outcomes

The primary outcome was the length of ICU stay among patients in the different groups (no sedation, propofol alone, sufentanil alone, and combination of propofol and sufentanil). Subsequently, factors associated with prolonged ICU stay were evaluated, such as trends in the ΔSOFA score, frequency of reintubation, onset of new pulmonary infiltrates, and incidence of AKI. ICU stay length was monitored for a period of 10 days from admission for each patient included in the study. Regarding the ΔSOFA score (calculated as the SOFA score on day X minus the SOFA score at hospital admission), values ≥2 were considered, starting from day 3 of the ICU stay. Similarly, the new onset of pulmonary infiltrates was monitored from day 3 to day 10. The incidence of reintubation was recorded from day 3 of ICU stay and included both patients initially admitted without intubation and those who, after being admitted were intubated and subsequently extubated and required reintubation due to respiratory failure. Finally, the incidence of AKI was assessed starting from day 3 in patients with no history of CKD.

### Ethical considerations and patient involvement

Ethical approval (number S-262/2023) was granted by the Ethical committee of the Witten/Herdecke University as this was a retrospective anonymous secondary data analysis. Consent forms were not required, given the retrospective study design. Patients were not involved in the formulation of the research question or outcome measures or in the designing or implementation of the study. They were also not consulted regarding the interpretation of data or manuscript writing.

### Statistical analyses

The data were entered into Excel sheets and imported into the Statistical Package for Social Sciences (SPSS) software (version 31.0, IBM Corp., Armonk, NY, USA) and GraphPad Prism 10.4.2 for data analysis and graphical representation of the results. ICU length of stay was analyzed as the outcome using Cox proportional hazards regression. ICU discharge was defined as the event, and patients still in the ICU on day 10 were censored at day 10. Sedation group was entered as the main predictor, with the no sedation group as the reference category. The multivariable model was adjusted for sex, DM, CKD, ASA class, surgical patient status, intubation on admission, need for noradrenaline, and COPD. Categorical variables were reported as counts and percentages. Differences between the groups across the assessed outcomes were evaluated using Fisher’s exact test. To evaluate whether the association between sedation strategy and outcome was independent of baseline differences between the groups, multivariable logistic regression analyses were performed for binary endpoints. Sedation group was entered as a categorical predictor with four levels (no sedation, propofol only, sufentanil only, and combination of propofol and sufentanil), using the no-sedation group as the reference category. Clinically relevant potential confounders were included in the models, including sex, ASA class, DM, COPD, CKD, surgical status, intubation at ICU admission, and need for noradrenaline at ICU admission. To improve model stability, selected covariates (ASA class, COPD, and CKD) were compressed into clinically meaningful categories before inclusion in the regression analyses. Results are presented as adjusted odds ratios (aORs) with 95% confidence intervals (CIs). A *p*-value <0.05 was considered statistically significant. Post-hoc power analysis was not conducted due to the limited sample size.

## Results

In total, 300 patients were included in the analysis and assigned to four groups based on sedation type: (a) no sedation (n = 100); (b) propofol only (n = 27); (c) combination of propofol and sufentanil (n = 74); (d) and sufentanil only (n = 99). Baseline characteristics are summarized in Table 1.

**Table 1.** Baseline characteristics and pathological medical history of the patient groups.

	No sedation n (%)	Sedation with propofol n (%)	Sedation with propofol + sufentanil n (%)	Sedation with sufentanil n (%)
Patients	100	27	74	99
Sex				
Male	55 (55)	13 (48.15)	42 (56.76)	63 (63.64)
Female	45 (45)	14 (51.85)	32 (43.24)	36 (36.36)
**Comorbidities**				
Diabetes mellitus	25 (25)	5 (18.52)	10 (13.52)	32 (32.32)
Chronic obstructive pulmonary disease	17 (17)	0 (0)	9 (12.16)	15 (15.15)
GOLD I	1 (1)	0 (0)	1 (1.35)	0 (0)
GOLD II	5 (5)	0 (0)	3 (4.05)	10 (10.10)
GOLD III	5 (5)	0 (0)	2 (2.70)	4 (4.04)
GOLD IV	6 (6)	0 (0)	3 (4.05)	1 (1.01)
Chronic kidney disease	22 (22)	1 (3.7)	5 (6.76)	8 (8.08)
G1	4 (4)	0 (0)	1 (1.35)	0 (0)
G2	5 (5)	0 (0)	1 (1.35)	4 (4.04)
G3a	1 (1)	0 (0)	0 (0)	1 (1.01)
G3b	4 (4)	1 (3.7)	0 (0)	0 (0)
G4	5 (5)	0 (0)	1 (1.35)	0 (0)
G5	3 (3)	0 (0)	2 (2.7)	3 (3.03)
**ASA classification**				
I	12 (12)	3 (11.11)	15 (20.27)	4 (4.04)
II	27 (27)	13 (48.15)	30 (40.54)	33 (33.33)
III	42 (42)	10 (37.04)	22 (29.73)	42 (42.42)
IV	19 (19)	1 (3.7)	7 (9.46)	19 (19.19)
V	0 (0)	0 (0)	0 (0)	1 (1.01)
**Surgical patients**				
Yes	30 (30)	22 (81.48)	61 (82.43)	61 (61.62)
No	70 (70)	5 (18.52)	13 (17.57)	38 (38.38)
**Reasons for ICU admission**				
**of surgical patients**				
RPM	26 (26)	22 (81.48)	61 (82.43)	59 (59.60)
CSM	0 (0)	0 (0)	0 (0)	2 (2.02)
CSC	4 (4)	0 (0)	0 (0)	0 (0)
**Intubated at ICU admission**				
Yes	10 (10)	24 (88.89)	64 (86.49)	76 (76.77)
No	90 (90)	3 (11.11)	10 (13.51)	23 (23.23)
**Need of noradrenalin on ICU admission**				
Yes	23 (23)	15 (55.56)	53 (71.62)	77 (77.78)
<0.25 µg/kg/min	21 (21)	11 (40.74)	47 63.51)	54 (54.55)
≥0.25 µg/kg/min	2 (2)	4 (14.81)	6 (8.11)	13 (13.13)
No	77 (77)	12 (44.44)	21 (28.38)	22 (22.22)

RPM: routine postoperative monitoring; CSM: closer surveillance after minor surgery; CSC: complicated surgical course; ICU: intensive care unit; ASA: American Society of Anesthesiologists

### Cumulative sedative doses in the sedation groups

**Figure 1.**
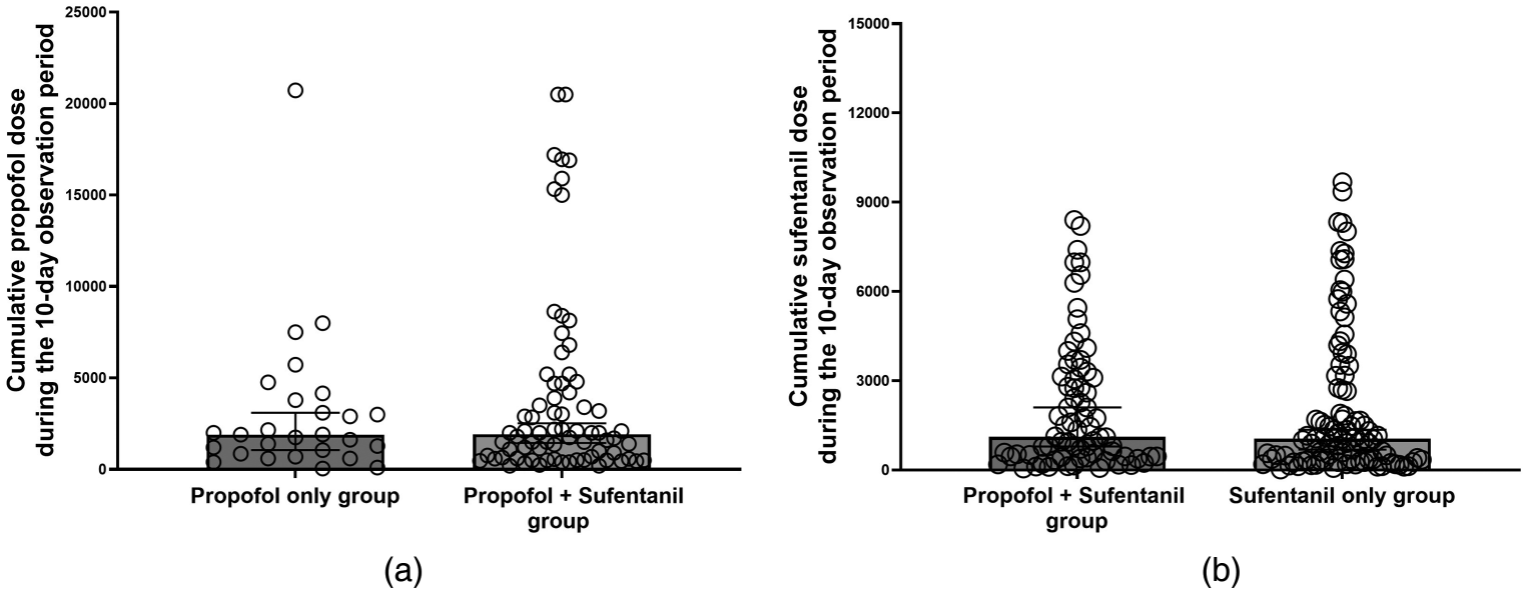
Comparison between cumulative propofol and sufentanil doses in the different sedation groups. The left graph shows only the cumulative propofol dose in the propofol only group and in the propofol plus sufentanil group, while the right panel shows only the cumulative sufentanil dose in the propofol plus sufentanil group and the sufentanil only group.

We first analyzed the cumulative doses of sedative administered during the first 10 days. Cumulative propofol doses were compared between the propofol only and propofol plus sufentanil groups, while cumulative sufentanil doses were compared between the sufentanil only and propofol plus sufentanil groups (Figure 1). Statistical comparison using the Mann–Whitney U test showed no significant differences in the cumulative propofol (*p* = 0.9195) or sufentanil (*p* = 0.5338) doses between the respective groups.

**Figure 2.**
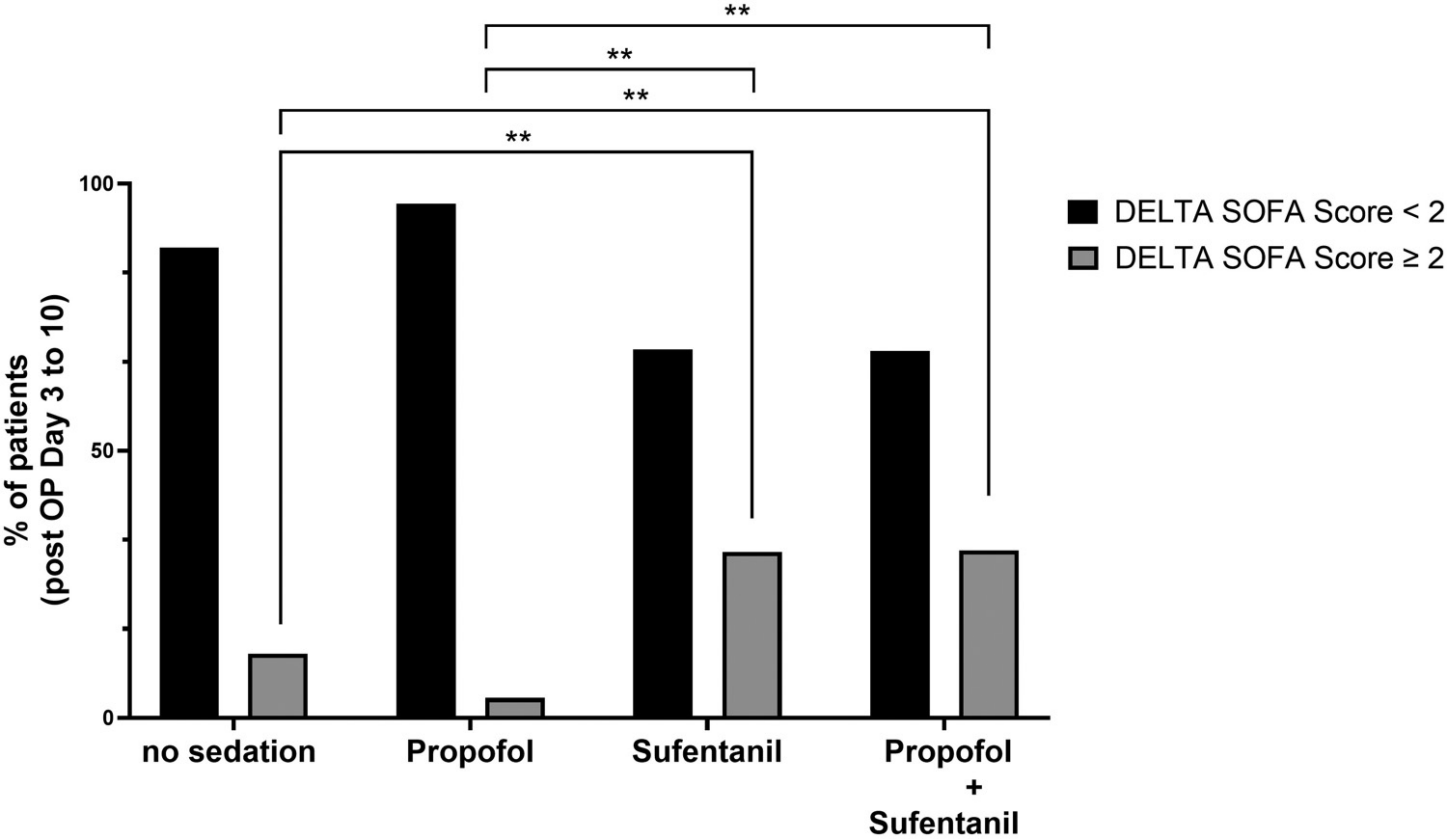
Percentage of patients with a ΔSOFA score ≥2 from days 3 to 10 of ICU stay. Bars represent the percentage of patients who developed the event “ΔSOFA score ≥2” in each group. A higher percentage of patients receiving sedation using sufentanil, with or without propofol, exhibit a ΔSOFA score ≥2 during a 7-day stay in the intensive care unit compared with patients who were not sedated and those who were administered only propofol for sedation. Data are presented as percentages. Differences in frequency were analyzed using Fisher’s exact test.

SOFA: Sequential Organ Failure Assessment; ICU: intensive care unit.

**Figure 3.**
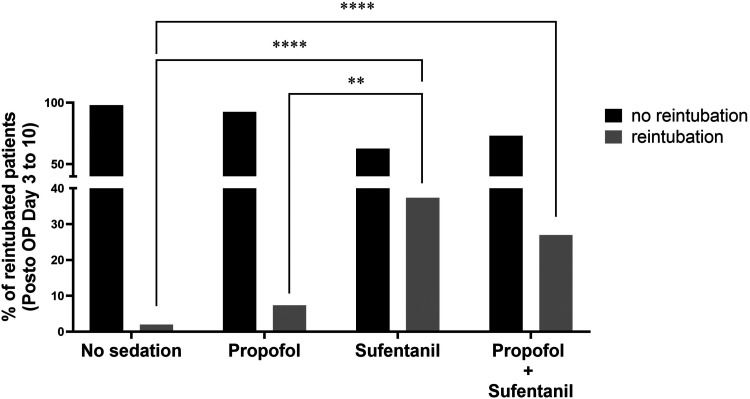
Frequency of reintubation. Bars represent the percentage of patients who developed the event “reintubation” in each group. Patients sedated with sufentanil showed a higher incidence of reintubation compared with those sedated using propofol alone and those who were not sedated. Statistical significance was assessed using Fisher’s exact test based on absolute counts. Data are presented as percentages. Differences in frequency were analyzed using Fisher’s exact test.

### Length of ICU stay

To investigate the association between the sedation type and length of ICU stay, multivariable Cox regression analysis of all 300 patients was performed. ICU discharge was defined as the event. During the 10-day observation period, 179 patients were discharged from the ICU, corresponding to 59.7% of the study population. The remaining 121 patients, corresponding to 40.3% of the study population, were censored on day 10 because they were still in the ICU. Sedation type was significantly associated with time to ICU discharge in the adjusted model. The global test for sedation type was statistically significant (*p* < 0.001). The no-sedation group served as the reference category. Compared with patients who were not sedated, those who were administered only propofol did not show a statistically significant difference in the probability of ICU discharge. The adjusted hazard ratio (aHR) was 0.619 (95% CI = 0.344–1.115, *p =* 0.110). In contrast, patients who were administered a combination of sufentanil and propofol had a significantly lower probability of ICU discharge compared with patients who were not sedated (aHR = 0.228, 95% CI = 0.129–0.403, *p* <0.001). Similarly, patients who were administered sufentanil only had a significantly lower probability of ICU discharge compared with patients who were not sedated (aHR = 0.267, 95% CI = 0.161–0.441, *p* < 0.001). Several potential confounders were included in the adjusted model: sex, DM, CKD, ASA class, surgical patient status, intubation on admission, need for noradrenaline, and COPD. Only Intubation on admission (hazard ratio (HR) = 1.606, 95% CI = 1.015–2.540, *p* = 0.043) and need for noradrenaline were significantly associated with time to ICU discharge (patients requiring noradrenaline < 0.25 µg/kg/min: HR = 0.617, 95% CI = 0.432–0.883, *p*-value = 0.008; patients requiring noradrenaline ≥ 0.25 µg/kg/min: HR = 0.311, 95% CI = 0.130–0.743, *p*-value = 0.009). The detailed results are reported in Table 2.

**Table 2.** Adjusted Cox regression analysis for time to ICU discharge according to sedation group

Variable	Adjusted HR	95% CI	*p*-value
Sedation group	-	-	<0.001^a^
Propofol only vs. no sedation	0.619	0.344–1.115	0.110
Propofol plus sufentanil vs. no sedation	0.228	0.129–0.403	<0.001^a^
Sufentanil only vs. no sedation	0.267	0.161–0.441	<0.001^a^
Male sex vs. female	0.811	0.597–1.103	0.182
Diabetes mellitus	1.348	0.947–1.920	0.098
Chronic kidney disease	0.693	0.444–1.083	0.108
ASA status classification	0.742	0.476–1.156	0.187
Surgical patient	0.991	0.708–1.388	0.959
Intubated on admission	1.606	1.015–2.540	0.043^b^
Need for noradrenaline	-	-	0.004^c^
Noradrenaline level <0.25 µg/kg/min	0.617	0.432–0.883	0.008^c^
Noradrenaline level ≥0.25 µg/kg/min	0.311	0.130–0.743	0.009^c^
COPD	1.419	0.928–2.169	0.106

ICU: intensive care unit; HR: hazard ratio; CI: confidence interval; ASA: American Society of Anesthesiologists; COPD: chronic obstructive pulmonary disease

### Delta SOFA score progression

From days 3–10 of ICU stay, the proportion of patients with a ΔSOFA score ≥2 was highest in the propofol plus sufentanil group (31.30%), followed by that in the sufentanil only group (31.10%). In contrast, this threshold was exceeded in only 3.7% of the patients in the propofol only group and in 12.0% of those who were not sedated. Statistically significant differences were observed between the sufentanil only group and both propofol only *(p = *0.0024) and no sedation (*p* = 0.001) groups. Similar differences were observed in the propofol plus sufentanil group compared with the propofol only *(p = *0.0033) and no sedation (*p* = 0.0023) groups (Figure 2). Results of the multivariable logistic regression are presented in Supplementary Table 1. Sedation type was significantly associated with the outcome, using the no-sedation group as the reference category (*p* < 0.001). Sedation using propofol alone was not significantly associated with the outcome, whereas sedation using a combination of propofol and sufentanil (aOR = 32.472, 95% CI = 9.460–111.466, *p* < 0.001) and sufentanil alone (aOR = 22.314, 95% CI = 7.629–65.269, *p* < 0.001) was associated with significantly higher odds of the outcome. Among the covariates, CKD, ASA status, intubation on admission, and need for noradrenaline showed significant associations.

### Reintubation rates

The frequency of reintubation between days 3 and 10 was highest among patients sedated using sufentanil alone (37.4%), followed by those sedated using a combination of propofol and sufentanil (27%). In contrast, only 7.4% of patients in the propofol only group and 2% in the no sedation group required reintubation. The difference was statistically significant between the sufentanil only group and both propofol only (*p* = 0.0021) and no sedation (*p* < 0.0001) groups. A significant difference was also observed between the propofol plus sufentanil group and no sedation group (*p* < 0.0001); however, the comparison with the propofol only group did not reach statistical significance (Figure 3). In the multivariable logistic regression model (Supplementary Table 2), sedation type was significantly associated with reintubation (*p* < 0.001), using the no-sedation group as the reference category. Compared with no sedation, propofol only sedation was associated with higher odds of reintubation (aOR = 20.978, 95% CI = 2.313–190.271, *p* = 0.007). Sedation using propofol plus sufentanil (aOR = 127.977, 95% CI = 22.001–744.419, *p* < 0.001) as well as sufentanil alone (aOR = 184.283, 95% CI = 33.768–1005.703, *p* < 0.001) was also associated with markedly higher odds of reintubation. Among the included covariates, intubation on admission was independently associated with lower odds of reintubation (aOR = 0.091, 95% CI = 0.033–0.249, *p* < 0.001). The remaining covariates were not significantly associated with reintubation in this model.

### Pulmonary infiltrates

**Figure 4.**
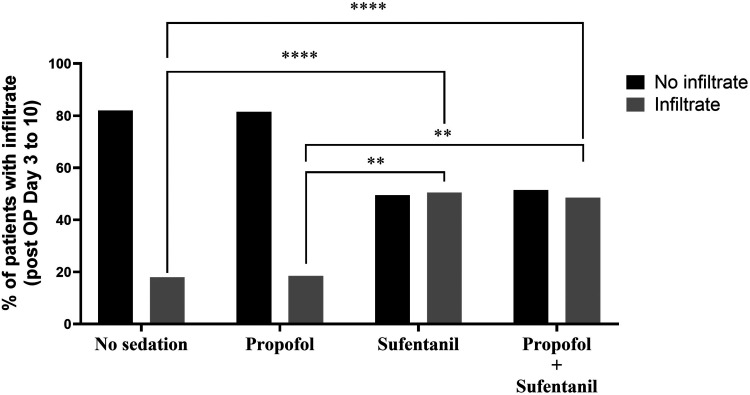
Onset of new pulmonary infiltrates. Bars represent the percentage of patients who developed the event “new pulmonary infiltrates” in each group. Patients sedated with a combination of sufentanil and propofol exhibited a higher incidence of pulmonary infiltrates between days 3 and 10 of intensive care unit stay compared with patients who were not sedated and those sedated with propofol alone. Statistical significance was assessed using Fisher’s exact test based on absolute counts. Data are presented as percentages. Differences in frequency were analyzed using Fisher’s exact test.

As shown in Figure 4, new pulmonary infiltrates were observed more frequently in patients who were administered only sufentanil for sedation (50.5%) and those administered a combination of propofol and sufentanil (48.6%) compared with those who were administered propofol alone (18.5%) and those who were not sedated (18%). The difference was statistically significant for the comparison of the sufentanil only group with the propofol only (*p* = 0.0039) and no sedation (*p* < 0.0001) groups. The propofol plus sufentanil group demonstrated a significant difference when compared with the no sedation (*p* < 0.0001) and propofol only (*p* = 0.0065) groups. In the multivariable logistic regression model, sedation type was significantly associated with the outcome pulmonary infiltrates (Supplementary Table 3), with no sedation used as the reference (*p* < 0.001). Sedation using only propofol was not significantly associated with the outcome (aOR = 2.451, 95% CI = 0.642–9.355, *p* = 0.190). In contrast, both sedation using propofol plus sufentanil (aOR = 8.731, 95% CI = 3.158–24.132, *p* < 0.001) and that using sufentanil alone (aOR = 7.076, 95% CI = 2.832–17.682, *p* < 0.001) were associated with significantly higher odds of the outcome. Among the covariates, sex, and CKD remained significant predictors.

**Figure 5.**
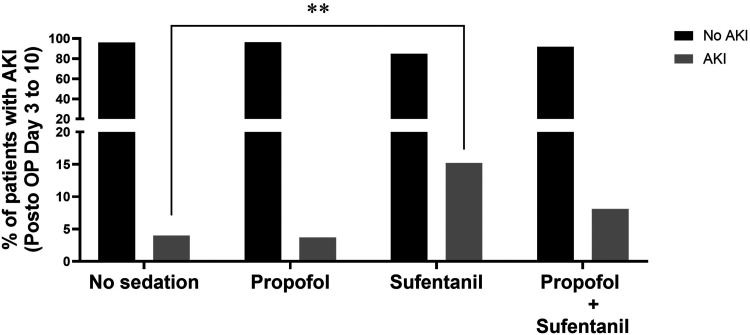
Frequency of acute kidney injury (AKI). Bars represent the percentage of patients who developed the event “AKI” in each group. Patients sedated with sufentanil alone exhibited a higher incidence of renal failure compared with the no sedation group. Statistical significance was assessed using Fisher’s exact test based on absolute counts. Data are presented as percentages. Differences in frequency were analyzed using Fisher’s exact test.

### Incidence of AKI

The incidence of AKI between days 3 and 10 was highest in the sufentanil only group (15.2%), followed by that in the propofol plus sufentanil group (8.1%) and propofol only group (3,7%); the incidence of AKI was 4% in the no sedation group. A statistically significant difference was observed only between the sufentanil only and no sedation groups (*p* = 0.0081), with other comparisons not reaching statistical significance (Figure 5). In the multivariable logistic regression analysis, sedation type was significantly associated with the outcome (AKI incidence, *p *= 0.049) (Supplementary Table 4). Propofol only sedation was not associated with a significant change in the odds of the outcome compared with no sedation (aOR = 2.461, 95% CI = 0.211–28.749, *p* = 0.473). Sedation using a combination of propofol and sufentanil was associated with higher odds of the outcome; however, this association narrowly missed reaching statistical significance (aOR = 5.018, 95% CI = 0.992–25.379, *p* = 0.051). Sufentanil only sedation was significantly associated with increased odds of the outcome compared with no sedation (aOR = 7.041, 95% CI = 1.716–28.896, *p* = 0.007). None of the included covariates reached statistical significance; however, the covariate of sex showed a nonsignificant trend toward higher aORs of the outcome.

## Discussion

In this retrospective cohort study of 300 critically ill adult ICU patients, sedation with sufentanil, either as monotherapy or in combination with propofol, was associated with significantly poorer short-term outcomes compared with propofol alone or no sedation. Patients who were administered sufentanil-based sedation exhibited longer ICU stays, higher ΔSOFA scores, higher reintubation rates, and an increased incidence of pulmonary complications and AKI. This observation aligns with previous evidence suggesting that certain sedative regimens, particularly those involving potent opioids such as sufentanil, contribute to delayed weaning from mechanical ventilation and prolonged critical care stays due to their cumulative pharmacologic effects and impact on respiratory drive and neurological recovery.^19–21^ Moreover, patients in the sufentanil and propofol sufentanil groups more frequently demonstrated a ΔSOFA score ≥2 during the observation period, indicating more severe and sustained organ dysfunction. We considered the ΔSOFA score rather than a fixed SOFA threshold because the ΔSOFA score captures changes in organ dysfunction relative to baseline.^22,23^ Our findings suggest that the use of sufentanil (alone or in combination with propofol) is associated with worse short-term organ function outcomes. The mechanisms could include immunosuppressive effects, respiratory complications, and hypotension related to high-dose opioid exposure.^24,25^ A particularly concerning finding was the higher incidence of reintubation and new pulmonary infiltrates among patients sedated using sufentanil alone. Reintubation is a known marker for respiratory failure and is associated with increased mortality, prolonged ventilation, and nosocomial infections.^26^ Pulmonary infiltrates, as observed in this study, further support the possibility of sedation-related pulmonary complications. In contrast, patients sedated using propofol alone showed more favorable outcomes across several parameters. Propofol has been associated with shorter ICU stays and faster neurological recovery due to its rapid onset and short duration of action, particularly when used in light sedation strategies.^27,28^ These characteristics may explain the comparatively lower ΔSOFA scores and reduced incidence of reintubation in the propofol group. In our analysis, patients sedated using sufentanil alone showed a slight increase in the incidence of AKI compared with those who were not sedated. Although the difference was modest, this trend may be clinically relevant, especially in critically ill patients who are already at an elevated risk of renal impairment. The underlying mechanisms could include opioid-induced hypotension or alterations in renal perfusion, both of which have been documented in previous studies examining the hemodynamic effects of high-dose opioids.^29^ Taken together, these data suggest that sedation with propofol alone is more favorable compared with sedation using sufentanil alone or in combination with propofol. This is in line with findings from a prospective randomized study in which propofol, when compared with sufentanil and midazolam, demonstrated the most favorable safety profile and was associated with a faster recovery time.^30^

In the present study, propofol only sedation was not associated with worse short-term outcomes compared with no sedation, whereas opioid-based sedation strategies, including sufentanil, were associated with less favorable short-term outcome patterns. Taken together, these findings suggest that sufentanil-based sedation is more unfavorable than propofol-based sedation in this clinical setting. However, this interpretation must be made with considerable caution. The propofol only group included only 27 patients, representing a sample size that is substantially smaller than that in the other study groups. This marked imbalance in group size limited the robustness of the comparisons and, importantly, made an appropriately adjusted multivariable assessment challenging as the corresponding regression estimates became unstable and unreliable for interpretation. Therefore, although the overall pattern of these data points toward a potentially less favorable effect of opioid-based sedation compared with propofol alone, the relatively small sample size of the propofol group represents a major study limitation. This size-based limitation and the resulting wide CIs of the OR estimates reduce the robustness and interpretability of our analyses. Therefore, a reliable post-hoc power analysis for this subgroup comparison was not considered feasible. Thus, the apparent advantage of propofol only sedation over sufentanil-based regimens should be considered hypothesis-generating rather than definitive, and confirmation in larger, more balanced cohorts is needed. The multivariable logistic regression analysis allowed adjustment for relevant clinical confounders, including comorbidities, surgical status, intubation on admission, and need for noradrenaline. After adjustment, the association between sedation strategy and the short-term outcomes remained significant, suggesting that the observed effect was not solely explained by these covariates. Nevertheless, given the retrospective design, residual confounding cannot be excluded, and the results should therefore be interpreted as associations rather than causal effects.

As reported in Table 1, one notable limitation of this study is the presence of a significant imbalance in the distribution of surgical versus nonsurgical patients across the four groups. Specifically, a higher proportion of surgical patients was observed in the three sedated groups, whereas nonsurgical patients were more prevalent in the no sedation group. This discrepancy introduces a potential confounding factor as undergoing surgery may negatively impact the postoperative outcomes independently of the type of sedation used. Despite this, no statistically significant differences were detected in the proportion of surgical patients among the sedated subgroups, those receiving propofol with or without sufentanil and those sedated using sufentanil alone, suggesting a more comparable distribution within the sedated cohorts. The reason for ICU admission among surgery patients was not included in the multivariable logistic regression analysis because several categories contained too few patients to allow a reliable statistical estimation. Regarding ASA physical status classification, the group sedated using a combination of propofol and sufentanil included a higher percentage of ASA I patients, indicating a slightly healthier baseline status in this subgroup. This variation could influence the interpretation of outcomes as ASA I patients generally present lower perioperative risk. In our cohort, most patients in the propofol only group were cardiac surgical patients who had undergone coronary artery bypass graft or ascending aortic replacement. These procedures are commonly associated with relevant bleeding risk and postoperative hemodynamic instability, and these patients frequently require vasopressor support. Thus, the propofol only group cannot be considered a particularly stable population selected because of lower hypotension risk. Notably, there were no cardiac surgical patients in the sufentanil only or propofol plus sufentanil groups. This makes it less likely that the observed associations were driven solely by a tendency to avoid propofol in unstable patients. We agree that relevant comorbidities, including cardiac conditions, may act as confounding factors in the association between sedation strategy and clinical outcome. Unfortunately, detailed information on specific cardiac comorbidities was not systematically collected in our retrospective dataset and could therefore not be included in the present analysis. Nevertheless, a major bias resulting from unmeasured cardiac disease appears unlikely to explain the observed findings. In our cohort, the propofol only group included the highest proportion of cardiac surgical patients, and may therefore have had the highest burden of underlying cardiac pathology. Accordingly, it is unlikely that a lower prevalence of cardiac disease in the propofol only group explains the more favorable outcome associated with the use of only propofol for sedation. In contrast, if anything, the concentration of cardiac surgical patients in the propofol only group would be expected to bias this group toward greater clinical complexity rather than toward an artificially improved prognosis. Therefore, we believe that the absence of systematically recorded cardiac comorbidities is an important limitation of the study; however, it is unlikely to fully explain the observed association between sedation type and outcome. Another important limitation of this study is the lack of a statistical correlation with the type and dosage of the different drugs used during general anesthesia induction across the various surgical specialties. It is well known, for example, that some teams still use high doses of opioids during cardiac surgery or other types of major surgery, and this may influence the postoperative course.^31^ Additionally, a higher proportion of patients with DM was identified in the group sedated using sufentanil alone. Given the known association between DM and adverse outcomes during ICU stays, this factor may have influenced group-specific results and should be considered when interpreting findings related to morbidity or recovery trajectories. In contrast, a greater proportion of patients with pre-existing CKD was observed in the no sedation group. However, the overall outcomes in this group do not appear to be disproportionately affected by this condition, suggesting that CKD did not exert a major confounding influence within the context of this study. Taken together, although several baseline imbalances exist among the study groups, particularly concerning surgical status, ASA class, and comorbidities such as diabetes and CKD, most of these differences are either statistically nonsignificant or unlikely to have critically biased the primary outcome. Nonetheless, these factors must be acknowledged as potential confounders and warrant further investigation in future prospective studies with more homogeneous group allocation.

Additional structural limitations of this study include the fact its retrospective design inherently limits the ability to establish causal relationships and increases the risk of selection bias and unmeasured confounders. Additionally, the relatively small number of patients in each group reduced the statistical power and limits the generalizability of the findings. Another significant limitation is the lack of data regarding infectious and hemodynamic parameters, which are crucial for fully understanding the clinical status and progression of critically ill patients. The absence of these variables restricts the ability to interpret the observed outcomes in the context of underlying physiological changes or infectious complications.

## Conclusions

This study highlights the potential impact of different sedation types on short-term outcomes in critically ill patients. Sedation using sufentanil, particularly when used alone, was associated with longer ICU stays, higher ΔSOFA scores, increased reintubation rates, and higher incidence of pulmonary complications. In contrast, propofol-based sedation appeared to be associated with more favorable clinical trajectories. These findings underscore the importance of carefully selecting and monitoring sedation regimens, emphasizing the need for individualized and minimal sedation approaches. Further prospective, randomized, and multicenter studies are warranted to confirm these associations and guide safer sedation practices in the intensive care setting. To confirm these findings and establish a potential association between sedation and poorer outcomes in ICU patients, prospective studies are required.

## Supplemental Material

sj-docx-1-imr-10.1177_03000605261461651 - Supplemental material for Impact of sedation with propofol and/or sufentanil on short-term outcomes in patients admitted to the intensive care unit: A retrospective study Supplemental material, sj-docx-1-imr-10.1177_03000605261461651 for Impact of sedation with propofol and/or sufentanil on short-term outcomes in patients admitted to the intensive care unit: A retrospective study by Luca Scarsella, Contrino Rita, El-Masri Dschamil and Thal Serge C in Journal of International Medical Research
