# Supplementary material for: Impact of sedation with propofol and/or sufentanil on short-term outcomes in patients admitted to the intensive care unit: A retrospective study
Source: J Int Med Res. 2026 Aug 10;54(8):03000605261461651. doi: 10.1177/03000605261461651 (PMC13474228; doi:10.1177/03000605261461651)
Supplement: Supplementary file 1 [file sj-docx-1-imr-10.1177_03000605261461651.docx]

**Supplementary Table 1**

| **Variable** | **Adjusted OR** | **95% CI** | **p-value** |
| --- | --- | --- | --- |
| Sedation group |  |  | <0.001*** |
| No sedation | Reference | Reference | — |
| Propofol only | 1.608 | 0.170–15.224 | 0.679 |
| Propofol + Sufentanil | 32.472 | 9.460–111.466 | <0.001*** |
| Sufentanil only | 22.314 | 7.629–65.269 | <0.001*** |
| Sex | 1.135 | 0.588–2.191 | 0.706 |
| Diabetes mellitus | 0.979 | 0.460–2.083 | 0.956 |
| Chronic kidney disease | 3.128 | 1.230–7.953 | 0.017* |
| ASA status | 2.562 | 1.080–6.079 | 0.033* |
| Surgical patients | 0.801 | 0.382–1.678 | 0.556 |
| Intubated on admission | 0.218 | 0.089–0.536 | <0.001*** |
| Need for noradrenaline |  |  | 0.009** |
| Need for noradrenaline, < 0.25 µg/Kg/min | 0.297 | 0.136–0.648 | 0.002** |
| Need for noradrenaline, ≥ 0.25 µg/Kg/min | 0.498 | 0.148–1.674 | 0.260 |
| COPD | 0.658 | 0.251–1.730 | 0.396 |

**Supplementary Table 2**

| **Variable** | **Adjusted OR** | **95% CI** | **p-value** |
| --- | --- | --- | --- |
| Sedation group (overall) |  |  | <0.001*** |
| No sedation | Reference | Reference | — |
| Propofol only | 20.978 | 2.313–190.271 | 0.007** |
| Propofol + Sufentanil | 127.977 | 22.001–744.419 | <0.001*** |
| Sufentanil only | 184.283 | 33.768–1005.703 | <0.001*** |
| Sex | 0.662 | 0.322–1.360 | 0.262 |
| Diabetes mellitus | 0.664 | 0.280–1.572 | 0.351 |
| Chronic kidney disease | 1.021 | 0.267–3.910 | 0.976 |
| ASA status | 2.198 | 0.774–6.244 | 0.139 |
| Surgical patients | 1.040 | 0.445–2.432 | 0.928 |
| Intubated on admission | 0.091 | 0.033–0.249 | <0.001*** |
| Need for noradrenaline (overall) |  |  | 0.972 |
| Need for noradrenaline, < 0.25 µg/Kg/min | 0.916 | 0.375–2.235 | 0.847 |
| Need for noradrenaline, ≥ 0.25 µg/Kg/min | 0.862 | 0.226–3.286 | 0.827 |
| COPD | 0.296 | 0.081–1.084 | 0.066 |

**Supplementary Table 3**

| **Variable** | **Adjusted OR** | **95% CI** | **p-value** |
| --- | --- | --- | --- |
| Sedation group |  |  | <0.001*** |
| No sedation | Reference | Reference | — |
| Propofol only | 2.451 | 0.642–9.355 | 0.190 |
| Propofol + Sufentanil | 8.731 | 3.158–24.132 | <0.001*** |
| Sufentanil only | 7.076 | 2.832–17.682 | <0.001*** |
| Sex | 2.142 | 1.237–3.707 | 0.007** |
| Diabetes mellitus | 0.977 | 0.524–1.820 | 0.942 |
| Chronic kidney disease | 2.631 | 1.112–6.223 | 0.028* |
| ASA status | 1.868 | 0.882–3.954 | 0.103 |
| Surgical patients | 0.642 | 0.343–1.204 | 0.167 |
| Intubated on admission | 0.744 | 0.341–1.626 | 0.459 |
| Need for noradrenaline |  |  | 0.464 |
| Need for noradrenaline, < 0.25 µg/Kg/min | 1.345 | 0.704–2.570 | 0.370 |
| Need for noradrenaline, ≥ 0.25 µg/Kg/min | 1.876 | 0.657–5.357 | 0.240 |
| COPD | 1.271 | 0.589–2.743 | 0.541 |

**Supplementary Table 4**

| **Variable** | **Adjusted OR** | **95% CI** | **p-value** |
| --- | --- | --- | --- |
| Sedation group |  |  | 0.049* |
| No sedation | Reference | Reference | — |
| Propofol only | 2.461 | 0.211–28.749 | 0.473 |
| Propofol + Sufentanil | 5.018 | 0.992–25.379 | 0.051 |
| Sufentanil only | 7.041 | 1.716–28.896 | 0.007** |
| Sex | 2.575 | 0.959–6.916 | 0.061 |
| Diabetes mellitus | 0.972 | 0.372–2.538 | 0.954 |
| Chronic kidney disease | 2.097 | 0.636–6.911 | 0.224 |
| ASA status | 1.876 | 0.670–5.249 | 0.231 |
| Surgical patients | 0.500 | 0.189–1.319 | 0.161 |
| Intubated on admission | 0.835 | 0.262–2.665 | 0.761 |
| Need for noradrenaline |  |  | 0.803 |
| Need for noradrenaline, < 0.25 µg/Kg/min | 0.804 | 0.278–2.323 | 0.687 |
| Need for noradrenaline, ≥ 0.25 µg/Kg/min | 1.232 | 0.250–6.058 | 0.798 |
| COPD | 1.250 | 0.399–3.915 | 0.701 |
